# Inoculation with *Bradyrhizobium ottawaense* Effectively Reduces N_2_O Emissions from Degraded Soybean Nodules in Andosols Dominated by *nosZ*-lacking Bradyrhizobia

**DOI:** 10.1264/jsme2.ME26022

**Published:** 2026-09-10

**Authors:** Misa Tsubokura, Shintaro Hara, Tsubasa Ohbayashi, Masanori Takeda, Yuma Sasaki, Haruka Ukita, Miho Fujimori, Kaori Kakizaki, Manabu Itakura, Kiwamu Minamisawa, Hiroko Akiyama

**Affiliations:** 1 Institute for Agro-Environmental Sciences, National Agriculture and Food Research Organization (NARO), Tsukuba, Ibaraki, 305–8604, Japan; 2 Graduate School of Life Sciences, Tohoku University, Sendai, Miyagi, 980–8577, Japan

**Keywords:** N_2_O mitigation, *Bradyrhizobium ottawaense*, N_2_O reductase gene (*nosZ*), soybean nodule, pot experiment

## Abstract

Nitrous oxide (N_2_O) is a potent greenhouse gas, and its largest anthropogenic source is agricultural soils. In these soils, N_2_O emissions are produced from nitrogen fertilization and nitrogen-rich crop residues, such as soybean nodules. Previous studies demonstrated that inoculation with *Bradyrhizobium diazoefficiens* possessing the N_2_O reductase gene (*nosZ*) effectively mitigated N_2_O emissions from soybean fields at harvest. More recently, *Bradyrhizobium ottawaense*, which exhibits higher *nosZ* expression, reduced N_2_O emissions more effectively than *B. diazoefficiens* during the degradation of soybean nodules formed under sterile vermiculite conditions. Although the effects of inoculation with *B. ottawaense* have subsequently been exami­ned under natural soil conditions, it remains unclear whether these effects are maintained across different soil types and soybean cultivars. Therefore, we herein investigated the effects of inoculation with *B. ottawaense* on its competitiveness and N_2_O emissions in pot experiments using two field soils (Andosol and Fluvisol) and three Japanese commercial soybean cultivars (‘Hatayutaka’, ‘Yukihomare R’, and ‘Satonohohoemi’). The results demonstrated that inoculation with *B. ottawaense* significantly increased higher nodule occupancy of the inoculant strain compared with the non-inoculated controls in the two field soils. Moreover, *B. ottawaense* tended to reduce N_2_O emissions in Andosol dominated by *nosZ*-lacking bradyrhizobia, regardless of the soybean cultivar. No significant N_2_O-reducing effects were observed in Fluvisol dominated by *nosZ*-possessing bradyrhizobia. Collectively, these results suggest the applicability of inoculation with *B. ottawaense* to diverse soybean cultivars and its effectiveness in mitigating N_2_O emissions from soybean fields in which indigenous *nosZ*-lacking bradyrhizobial populations are dominant, such as Andosols.

Nitrous oxide (N_2_O) is a potent greenhouse gas with a global warming potential that is 273-fold higher than that of carbon dioxide (Forster *et al.*, 2021). It is also an ozone-depleting substance (WMO, 2022). Approximately 58% of global anthropogenic N_2_O emissions are due to agricultural activities (Tian *et al.*, 2024), including the application of synthetic fertilizers and manure (Xia *et al.*, 2020; Menegat *et al.*, 2022), and the decomposition of crop residues with a low carbon-to-nitrogen ratio, such as leguminous crops (Huang *et al.*, 2004; Rochette *et al.*, 2004; Akiyama *et al.*, 2020). In agroecosystems, N_2_O is primarily produced via microbial nitrification and denitrification, which are regulated by soil environmental factors (*e.g.*, nitrogen content, moisture, and pH) (Wrage *et al.*, 2001; Wang *et al.*, 2021a). Consequently, various approaches have been proposed to mitigate N_2_O emissions, including optimizing nitrogen applications, utilizing enhanced-efficiency fertilizers, improving drainage, and adjusting soil pH through liming (Hassan *et al.*, 2022; Akiyama, 2024). Nevertheless, atmospheric N_2_O concentrations continue to increase (Tian *et al.*, 2024), highlighting the urgent need to develop innovative mitigation techniques.

Soybean (*Glycine max* [L.] Merr.) forms root nodules colonized by a group of nitrogen-fixing bacteria in the genus *Bradyrhizobium*, which supply ammonia fixed from atmospheric nitrogen. In the soybean rhizosphere, N_2_O emissions occur from degraded nodules during the harvest season (Rochette *et al.*, 2004; Yang and Cai, 2005; Inaba *et al.*, 2012; Akiyama *et al.*, 2016), which are mainly driven by denitrification via microorganisms, including soybean-nodulating bradyrhizobia and soil fungi (Inaba *et al.*, 2012; Moriuchi *et al.*, 2025). Denitrification is a sequential reduction process progressing from NO_3_^–^/NO_2_^–^ to N_2_O or N_2_ (Yoon *et al.*, 2019), and N_2_O is reduced to N_2_ by N_2_O reductase encoded by the *nosZ* gene (Zumft, 1997). Some bradyrhizobial strains lack the *nosZ* gene (*nosZ*– bradyrhizobia), and their abundance may be associated with net N_2_O emissions in the soybean rhizosphere (Uchida and Akiyama, 2013). In Japan, the population of *nosZ*– bradyrhizobia is abundant in Andosols (volcanic ash soils), which cover approximately half of upland fields (Shiina *et al.*, 2014; Shinjo and Takata, 2021). Accordingly, inoculation with bradyrhizobia possessing the *nosZ* gene (*nosZ+* bradyrhizobia), such as *Bradyrhizobium diazoefficiens*, has been proposed as an effective option to mitigate N_2_O emissions in soybean fields. These mitigating effects have been demonstrated in field studies conducted in Japan as well as in South America and France (Itakura *et al.*, 2013; Akiyama *et al.*, 2016; Melissa *et al.*, 2022; Hénault *et al.*, 2022).

In recent years, *nosZ+* bradyrhizobia have been identified within *Bradyrhizobium ottawaense* (Wasai-Hara *et al.*, 2020, 2023). Several strains of *B. ottawaense* isolated from sorghum roots were shown to exhibit higher N_2_O-reducing activity than wild-type *B. diazoefficiens* USDA110^T^ (Wasai-Hara *et al.*, 2023; Itakura and Minamisawa, 2026). Moreover, *B. ottawaense* was found to be capable of nodulating a wide range of commercial soybean cultivars (Minakata *et al.*, 2023; Win *et al.*, 2023, 2024), and soybean plants inoculated with *B. ottawaense* showed 43–57% nodule occupancy under field conditions (Hara *et al.*, 2024). Nishida *et al.* (2025) further demonstrated that incompatibility-bypassing strains of *B. ottawaense*, combined with soybean lines bred for enhanced host selectivity, achieved >60% nodule occupancy of the inoculant, with significant N_2_O reduction. Therefore, *B. ottawaense* is expected to be a highly potent bradyrhizobial species that reduces N_2_O emissions from soybean fields.

To confirm this potential, it was necessary to investigate the competitiveness and N_2_O-reducing effects of *B. ottawaense* across different soil types and commercial soybean cultivars. Therefore, we conducted pot experiments: (i) to identify the strain with superior N_2_O-reducing activity (Experiment 1); (ii) to examine its inoculation effects in two major soils of Japan (Experiment 2); and (iii) to evaluate its performance in three commercial soybean cultivars developed in Japan (Experiments 3 and 4).

## Materials and Methods

### *Bradyrhizobium* strains and culture conditions

The *Bradyrhizobium* strains used in the present study are listed in Table 1. *B. ottawaense* strains were isolated from diverse locations and host plants, while the S110 group (mixed strains of *B. diazoefficiens* isolated from soybean nodules in Hokkaido) was selected to cover a wide range of genomic diversity based on a comparative genomic anal­ysis (data not shown). *B. diazoefficiens* strain TSU1-4 (previously described as ‘TS1-4’ by Shiina *et al.*, 2014 and Hara *et al.*, 2024), which is phylogenetically close to *B. diazoefficiens* USDA110^T^, was isolated from a soybean nodule in Ibaraki. All strains were cultured at 30°C with agitation at 110 rpm in HM broth (Cole and Elkan, 1973) supplemented with 1 g L^–1^ arabinose and 0.25 g L^–1^ yeast extract.

### Pot experimental designs

Four pot experiments (Experiments 1–4) were performed in greenhouses under natural light at the Institute for Agro-Environmental Sciences, National Agriculture and Food Research Organization (NIAES, NARO) in Tsukuba, Ibaraki, Japan (36°0′N, 140°1′E). Two agricultural soils: Andosol and Fluvisol (also called Gray lowland soil in the Soil Classification System in Japan) were collected from the experimental fields at NIAES and sieved through a 4.75-mm mesh. The properties of Andosol and Fluvisol were previously described by Akiyama *et al.* (2020) and Toyoda *et al.* (2024), respectively. In Experiments 1 and 2, soils were filled into biodegradable Jiffy pots (approximately 0.3 L; Jiffy International AS), which were then placed in 1/5,000 a Wagner pots filled with vermiculite. In Experiment 3, 3 L of Andosol was directly filled into 1/5,000 a Wagner pots. These experiments used two Japanese commercial soybean cultivars: ‘Hatayutaka’ (medium-maturing) and ‘Yukihomare R’ (early-maturing), which were developed for the central (Kanto) and northern (Hokkaido) regions of Japan, respectively.

#### Experiment 1:

Experiment 1 consisted of two independent evaluations for analyzing nodule occupancy (Experiment 1-1, Fig. 1A) and measuring N_2_O flux (Experiment 1-2, Fig. 1B). These evaluations were conducted to compare the inoculation effects of eight *B. ottawaense* strains (SF21, SG09, SG10, SG23, SH12, FA1, FY1, or FY2) with *B. diazoefficiens* TSU1-4 in Andosol.

##### Experiment 1-1:

Dry soybean seeds (cv. Hatayutaka) were covered with moist vermiculite at room temperature for 1 day for pre-germination, and four seeds were then sown in the biodegradable pots. One milliliter of a bacterial suspension (1×10^9^‍ ‍cells‍ ‍mL^–1^) containing a strain of *B. ottawaense* or *B. diazoefficiens*, or sterile deionized water (as a non-inoculated control) was applied to each seed. Each treatment was performed in four replicates. At 13 days after sowing (DAS), one seedling was left per pot by thinning. At 37 DAS, shoot decapitation was performed. The nodules were counted and weighed per pot. They were then immediately frozen using liquid nitrogen and stored at –80°C until the PCR anal­ysis.

##### Experiment 1-2:

The pot preparation and bradyrhizobial inoculation were performed as described in Experiment 1-1. The seedlings were thinned at 10 DAS. Shoot decapitation was performed at 41 DAS, and the shoot parts were then dried and weighed. Gas sampling was conducted for 28 days after decapitation in the greenhouses.

#### Experiment 2:

Experiment 2 was conducted to examine differences in the inoculation effect of *B. ottawaense* SG09 between two field soils (Fig. 2A). Four dry soybean seeds (cv. Hatayutaka) were sown in biodegradable pots containing Andosol or Fluvisol, and the inoculation with *B. ottawaense* SG09 was then performed as described above. Each treatment was performed in ten replicates. Two of the four seedlings were thinned per pot at 7 DAS. Shoot decapitation was conducted at 31 DAS, and the shoot parts were then dried and weighed. The nodules in five of the ten pots were counted, weighed, and then stored until the PCR anal­ysis as described in Experiment 1-1. Nodule occupancy rates were investigated by *nosZ* multiplex PCR and an ITS-RFLP anal­ysis in three of the five pots. Gas sampling of the five other pots was conducted for 20 days in the greenhouses.

#### Experiment 3:

Experiment 3 was conducted to evaluate the host cultivar-dependent effects of the inoculation with *B. ottawaense* SG09 (Fig. 3A). The inoculation effect of SG09 was compared with those of *B. diazoefficiens* TSU1-4 and the S110 group. Four dry soybean seeds (cv. Hatayutaka or cv. Yukihomare R) were sown in 1/5,000 a Wagner pots containing Andosol, and the bradyrhizobial inoculation was then performed as described above. Each treatment was conducted in eight replicates. Two of the four seedlings were thinned at 14 DAS. Shoot decapitation was performed at 46 DAS, and the shoot parts were then dried and weighed. The nodules in four of the eight pots were counted, weighed, and stored until the PCR anal­ysis as described in Experiment 1-1. Nodule occupancy rates were subsequently investigated by *nosZ* multiplex PCR. Gas sampling of the four other pots was conducted for 45 days in the greenhouses.

Further details of methods and a description of Experiment 4 are provided in the Supplementary Materials.

### Analysis of nodule occupancy by *nosZ* multiplex PCR and the ITS-RFLP anal­ysis

The nodule occupancy rates of inoculated *B. diazoefficiens* and *B. ottawaense* carrying the *nosZ* gene were assessed by species-specific multiplex PCR, as previously described (Hara *et al.*, 2024). Briefly, DNA templates were prepared from crushed nodules in 96-well plates using crude extraction and a proteinase K treatment. Multiplex PCR was conducted with *nosZ*-specific primer sets (nosZ_BD-F/R for *B. diazoefficiens* and nosZ_BW-F/R for *B. ottawaense*; Table S1). In the RFLP anal­ysis of the 16S-23S rRNA gene internal transcribed region (the ITS-RFLP anal­ysis), PCR was conducted using ITS primers (BraITS-F and BraITS-R; Table S1) to simultaneously detect *Bradyrhizobium japonicum* and *Bradyrhizobium elkanii* species, as previously described (Saeki *et al.*, 2006; Itakura *et al.*, 2026), and the PCR products were subjected to RFLP typing with the restriction enzyme MspI (TaKaRa Bio).

### N_2_O flux measurement

N_2_O fluxes were measured every 3–4 days after decapitation. Gas sampling was always conducted at 9–10 a.m. to minimize diurnal fluctuations (Alves *et al.*, 2012). Headspace gases (30 mL) from each pot were collected using a 50-mL syringe at 0, 20, and 40 min after sealing with acrylic lids equipped with syringe septa. Samples were immediately injected into evacuated 15-mL vials. N_2_O concentrations were analyzed using a gas chromatograph (GC-2014; Shimadzu; Sudo and Yamamoto, 2017), in combination with a CH_4_-doped ^63^Ni electron capture detector at 340°C using a headspace auto-sampler (AOC-5000; Shimadzu; Sudo and Yamamoto, 2017). Nitrogen was used as the carrier gas. Fluxes were calculated from the linear change in N_2_O concentrations across the three time points (Hou *et al.*, 2016), while cumulative N_2_O emissions were calculated using the trapezoidal integration method.

### Statistical anal­ysis

All statistical anal­yses were conducted using R software (version 4.3.1) (R Core Team, 2023). *P* values <0.05 were considered to be significant. The statistical methods used are indicated in each figure legend, and a detailed anal­ysis method is provided in the Supplementary Materials.

### Genomic sequence accession numbers

The 16S rRNA gene sequences of the S110 group were deposited in the DDBJ Sequence Read Archive under accession numbers SAMD01705412–SAMD01705421 (Table 1).

## Results

### Experiment 1: N_2_O-reducing potential of eight *B. ottawaense* strains

In Experiment 1-1, the inoculations with each of the eight *B. ottawaense* strains and with *B. diazoefficiens* showed the significantly higher occupancy of *nosZ*+ bradyrhizobial inoculants, respectively, than the non-inoculated control (Fig. S2A, Table 2). On the other hand, the *nosZ*– bradyrhizobial population was dominant in the non-inoculated control. In Experiment 1-2, cumulative N_2_O emissions were significantly lower with the inoculations with each *B. ottawaense* strain (71.5–87.9% reductions) than with the non-inoculation (Fig. 1C and S2B, Table 2). No significant differences were observed in nodule occupancy or N_2_O reduction rates among the *B. ottawaense* strains and *B. diazoefficiens* strain. There were also no significant differences in the number and fresh weight of nodules or shoot dry weight among the treatments (Fig. S2C, D, and E). Soybean plants reached the reproductive stage at the time of decapitation regardless of the bradyrhizobial inoculation in both experiments. Collectively, these results demonstrate that the eight *B. ottawaense* strains used in this study exhibited similar capabilities for nodulation and N_2_O reduction.

Of these strains, various features of *B. ottawaense* SG09 have been extensively exami­ned, such as nodule formation, N_2_O-reducing activity, and the mechanisms underlying attachment to plant roots (Wasai-Hara *et al.*, 2023; Win *et al.*, 2023, 2024; Hara *et al.*, 2024; Toyoda *et al.*, 2024; Nishida *et al.*, 2025; Takeguchi *et al.*, 2025; Itakura and Minamisawa, 2026). Therefore, strain SG09 was selected for subsequent experiments.

### Experiment 2: Effects of the *B. ottawaense* inoculation on N_2_O emissions from degraded soybean nodules in Andosol and Fluvisol

The nodule occupancy rates of *B. ottawaense* in the SG09-inoculated treatment were 63.3 and 83.0% in Andosol and Fluvisol, respectively (Fig. 2B and C, Table 2). In contrast, almost no occupancy of *B. ottawaense* was observed in the non-inoculated control in either soil, and the predominant bradyrhizobial species were *B. elkanii* (*nosZ*–) (71.7%) in Andosol and *B. diazoefficiens* (59.6%) in Fluvisol (Fig. 2C and S3, Table 2).

Maximum N_2_O fluxes in Andosol were 47.6±25.3 and 148.1±131.7 μg N_2_O-N m^–2^ h^–1^ from the non- and SG09-inoculated pots, respectively, at 10 days after decapitation (Fig. S4A). Cumulative N_2_O emissions were 58.6% lower with the SG09 inoculation than with the non-inoculation (Table 2), although this difference was not significant due to large variations in the non-inoculated treatment (Fig. 2D). N_2_O fluxes were markedly lower in Fluvisol than in Andosol regardless of the bradyrhizobial inoculation, and no significant difference was observed in cumulative N_2_O emissions between the non- and SG09-inoculated treatments (Fig. 2E, Table 2). A two-way ANOVA of cumulative N_2_O emissions revealed a significant effect of soil type (*P*=0.024), but not of bradyrhizobial inoculation (*P*=0.093). No interaction was detected between the two factors (*P*=0.124).

Shoot dry weight and nitrogen content in both soils were similar with the SG09 inoculation and non-inoculation (Fig. S4B and C). However, in Fluvisol, SG09-inoculated soybean plants formed significantly more nodules with a greater fresh weight than non-inoculated plants, whereas no significant differences were observed in Andosol (Fig. S4D and E). Furthermore, the developmental stages of plants were the same in both soils (vegetative stage) at the time of decapitation. These results indicate that the SG09-inoculated treatment had no effect on plant growth.

### Experiment 3: Effects of the *B. ottawaense* inoculation on N_2_O emissions in different soybean cultivars

The nodule occupancy rates of *B. ottawaense* were 59.2 and 28.3% in ‘Hatayutaka’ and ‘Yukihomare R’, respectively, under the SG09-inoculated treatment (Fig. 3B and C, Table 2). In contrast, the occupancy of *B. ottawaense* was negligible in both cultivars in the non-, TSU1-4-, and S110-inoculated treatments (0.0–5.8%). The TSU1-4- and S110-inoculated treatments increased the occupancy of *B. diazoefficiens* in both cultivars significantly more than the respective non-inoculated controls (45.0–76.7%).

Cumulative N_2_O emissions in non-inoculated treatments differed between soybean cultivars: soils cultivated with ‘Yukihomare R’ showed 1.6-fold higher emissions than those cultivated with ‘Hatayutaka’ (Fig. 3D and E, Table 2). Despite this difference, the SG09-, TSU1-4-, and S110-inoculated treatments reduced N_2_O emissions from each soil cultivated with ‘Hatayutaka’ and ‘Yukihomare R’ significantly more than their respective non-inoculated controls (Fig. 3D, 3E, S5A, and S5B). The reduction rates of these *nosZ*+ bradyrhizobial-inoculated treatments were similar between the two cultivars (Table 2). The number of nodules was higher in ‘Yukihomare R’ than in ‘Hatayutaka’, and the TSU1-4-inoculated treatment tended to promote nodule formation more than the other treatments in both cultivars (Fig. S6A and B). A two-way ANOVA showed the significant effects of soybean cultivar (*P*<0.0001) and bradyrhizobial inoculation (*P*=0.0127) on the number of nodules, with no interaction (*P*=0.539). The developmental stages of the two cultivars at decapitation differed regardless of the inoculants: ‘Hatayutaka’ was at the vegetative stage, while ‘Yukihomare R’ had reached the reproductive stage. However, shoot dry weight was not significantly affected by the bradyrhizobial inoculation (Fig. S6C and D).

## Discussion

All experiments in the present study showed that the inoculation with *B. ottawaense* resulted in significantly higher nodule occupancy than with the non-inoculated controls (Table 2). However, the N_2_O-reducing effects of *B. ottawaense* may vary depending on soil type. Strain SG09 tended to reduce N_2_O emissions in Andosol, but not in Fluvisol (Fig. 2D and E). This variation may be explained by differences in the abundance of indigenous *nosZ*+ bradyrhizobia between the two soils. In agricultural fields of Japan, *nosZ*– bradyrhizobia dominate Andosols with a porous structure and lower bulk density, whereas *nosZ*+ bradyrhizobia dominate alluvial soils with a dense structure, such as Fluvisols (Shiina *et al.*, 2014; Akiyama *et al.*, 2020; Takata *et al.*, 2021). Since *nosZ*+ bradyrhizobia performing N_2_O respiration—most of which belong to *B. diazoefficiens* commonly found in agricultural soils—are frequently detected under anaerobic conditions that develop in fine-textured soils (Saeki *et al.*, 2013), Fluvisols are likely to have an advantage in their survival. In the present study, since N_2_O emissions were minimal in Fluvisols where indigenous *nosZ*+ bradyrhizobia were already abundant, the N_2_O-reducing effects of SG09 appeared to be limited. Therefore, inoculation with *B. ottawaense* may be effective in reducing N_2_O emissions from soybean fields with a low abundance of indigenous *nosZ*+ bradyrhizobia.

In addition, cumulative N_2_O emissions were higher in the treatments using ‘Yukihomare R’ than in those using ‘Hatayutaka’ (Fig. 3D and E, Table 2), which may be attributed to differences in nodule number. Nodule number is a key factor affecting N_2_O production (Kim *et al.*, 2005) and markedly increases after the reproductive stage (Weil and Ohlrogge, 1975; Takahashi *et al.*, 2005; Arachchige *et al.*, 2020). ‘Yukihomare R’ reached the reproductive stage earlier and formed more nodules than ‘Hatayutaka’ (Fig. S6A and B), which may have contributed to higher N_2_O production. However, the N_2_O-reducing effects of SG09 were consistent regardless of differences in N_2_O emission levels. Therefore, inoculation with *B. ottawaense* has the potential to achieve a stable N_2_O reduction across diverse soybean cultivars.

In Experiment 4, the supplemental experiment using ‘Hatayutaka’ and ‘Satonohohoemi’ (Fig. S7 and S8), N_2_O-reducing effects were less pronounced than in Experiment 3, despite the high occupancy of *nosZ+* bradyrhizobia (Table S2). N_2_O emission levels in the non-inoculated controls of Experiment 4 were also markedly lower than in the other experiments, regardless of the cultivar (Fig. S5C and D). Only Experiment 4 was conducted during winter and had the lowest solar radiation (Table S3). The low light availability during this period may have restricted soybean development, including nodule formation (Wang *et al.*, 2021b), resulting in fewer nodules in Experiment 4 (Fig. S6A, S6B, S8A, and S8B). Consequently, the N_2_O-reducing effects of *nosZ+* bradyrhizobia may not be detectable under extremely low N_2_O emission levels due to restricted nodule development.

*B. ottawaense* SG09 possesses two transcription start sites within the *nosR* transcriptional organization under N_2_O-respiring conditions (Wasai-Hara *et al.*, 2023). In addition, this strain exhibits a markedly higher maximum N_2_O reduction rate than *B. diazoefficiens* USDA110^T^, which possesses a single transcription start site, thereby contributing to superior N_2_O-reducing activity observed under both pure liquid culture and sterile soil conditions (Itakura and Minamisawa, 2026). However, the present study using natural soils showed no significant difference in N_2_O-reducing effects between *B. ottawaense* and *B. diazoefficiens* (Fig. 1C, 3D, 3E, S7D, and S7E). A similar discrepancy was reported in previous studies, in which the N_2_O-reducing effects of *nosZ*-carrying bacteria were often less effective under soil conditions than under pure culture conditions (Itakura *et al.*, 2013; Usyskin-Tonne *et al.*, 2019). Although the mechanisms underlying this discrepancy remain unclear, it may be attributed to differences between the free-living state in liquid cultures and the bacteroid state within soybean nodules. Further studies are needed to elucidate and overcome the challenges associated with scaling up from pure culture systems to soil environments.

The N_2_O-reducing effects of *nosZ*+ bradyrhizobia may be affected by soil moisture. Denitrification-derived N_2_O production generally increases with the water-filled pore space (WFPS) of soil, particularly between 50–80% (Davidson, 1993). In the present pot study and previous field studies, inoculation with *nosZ+* bradyrhizobia mitigated N_2_O production under the relatively stable WFPS conditions typically associated with denitrification-derived N_2_O production (WFPS: >40–50%, Fig. S9; Itakura *et al.*, 2013; Akiyama *et al.*, 2016). On the other hand, a recent field study reported no significant N_2_O reduction following inoculation with *B. ottawaense* under field conditions with large fluctuations in WFPS (WFPS: 26–71%, Toyoda *et al.*, 2024). Therefore, the effects of soil moisture on bradyrhizobial N_2_O reduction warrant further study.

In conclusion, inoculation with *B. ottawaense* significantly increased nodule occupancy and tended to reduce N_2_O emissions in multiple commercial soybean cultivars under pot-scale soil conditions. Moreover, inoculation with *B. ottawaense* may be more effective in reducing N_2_O emissions from Andosol, in which *nosZ*– bradyrhizobia are dominant, than from Fluvisol, in which *nosZ*+ bradyrhizobia are dominant. On the other hand, in the present study, the N_2_O-reducing effects of *B. ottawaense* were similar to those of *B. diazoefficiens*, which is in contrast to the findings of a previous laboratory-scale study (Wasai-Hara *et al.*, 2023). To improve the reliability of bradyrhizobial N_2_O mitigation in agricultural fields, further research is needed to assess the N_2_O-reducing potential of *B. ottawaense* under various soil conditions that reflect field environments.

## Acknowledgements

The authors thank Yeonghoo Kim for his help with sampling and analyzing the gas, and Mariko Nakagawa and Hikaru Uno for their help with preparing the experimental tools. We are also grateful to the technical support staff of NARO for their support in conducting the experiments.

### Conflicts of Interest

The authors declare that there are no conflicts of interest.

### Sources of Funding

The present study was supported by the Moonshot project JPNP18016, commissioned by New Energy and Industrial Technology Development Organization (NEDO).

## Citation

Tsubokura, M., Hara, S., Ohbayashi, T., Takeda, M., Sasaki, Y., Ukita, H., et al. (2026) Inoculation with *Bradyrhizobium ottawaense* Effectively Reduces N_2_O Emissions from Degraded Soybean Nodules in Andosols Dominated by *nosZ*-lacking Bradyrhizobia. *Microbes Environ ***41**: ME26022.

https://doi.org/10.1264/jsme2.ME26022

## Supplementary Material

Supplementary Material

## Figures and Tables

**Fig. 1.**
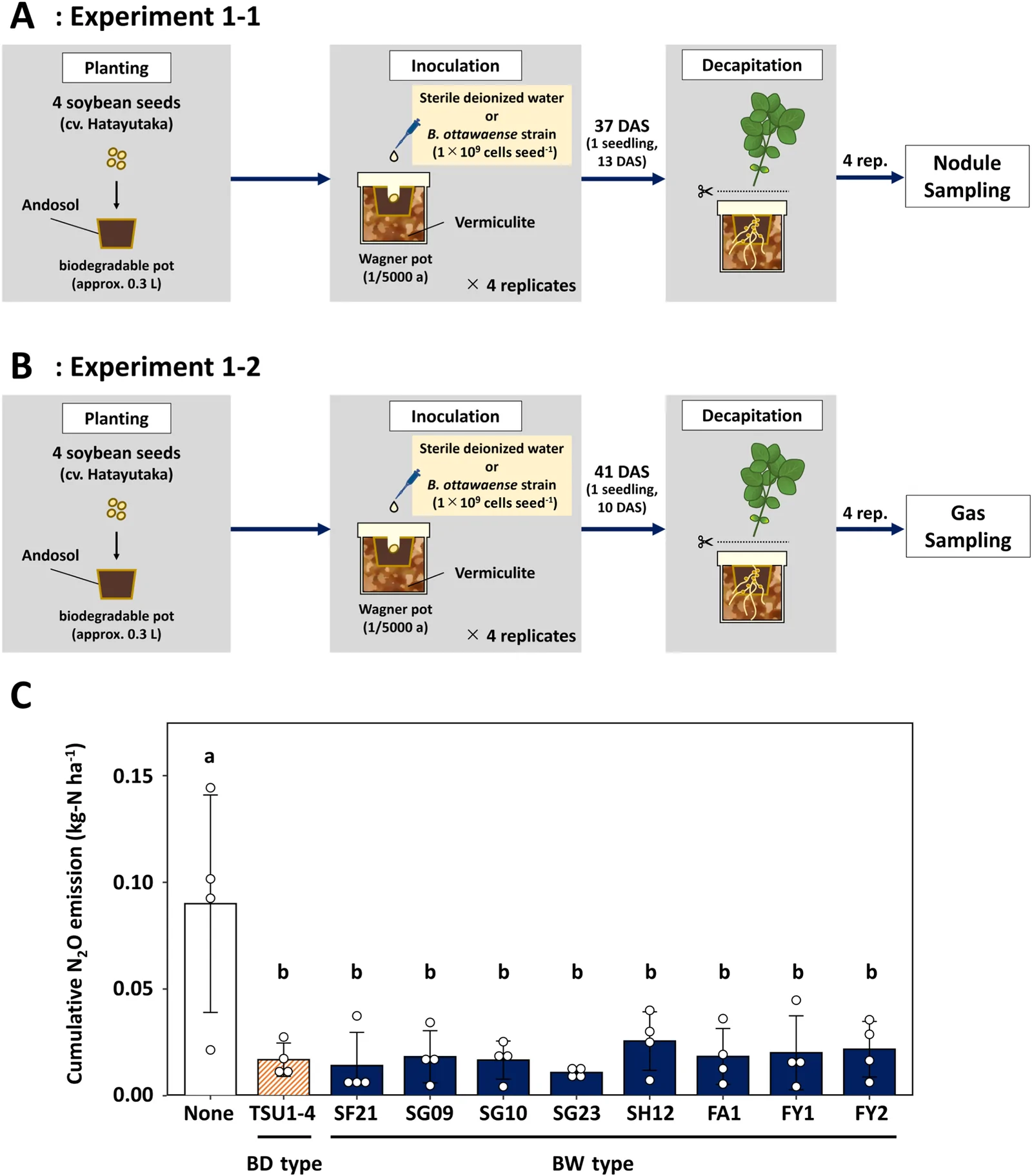
Effects of bradyrhizobial inoculations on nodule occupancy and N_2_O emission in Experiment 1. (A) Overview of Experiment 1. Treatments are non-inoculation (None), inoculation with *B. diazoefficiens* strain (TSU1-4) or *B. ottawaense* strain (any of SF21, SG09, SG10, SG23, SH12, FA1, FY1, or FY2). (A) Overview of Experiment 1-1 for analyzing nodule occupancy. DAS: days after sowing. (B) Overview of Experiment 1-2 for monitoring N_2_O emissions. (C) Cumulative N_2_O emissions from Andosol for 28 days from shoot decapitation. “BD type” and “BW type” indicate “*Bradyrhizobium diazoefficiens*” and “*Bradyrhizobium ottawaense*”, respectively. Values are means±SD (*n*=4 replicate pots). Different characters indicate significant differences (*P*<0.05); the significance of differences was evaluated using Tukey’s HSD test. Nodule occupancy rates by *nosZ*+ bradyrhizobia are shown in Fig. S2A.

**Fig. 2.**
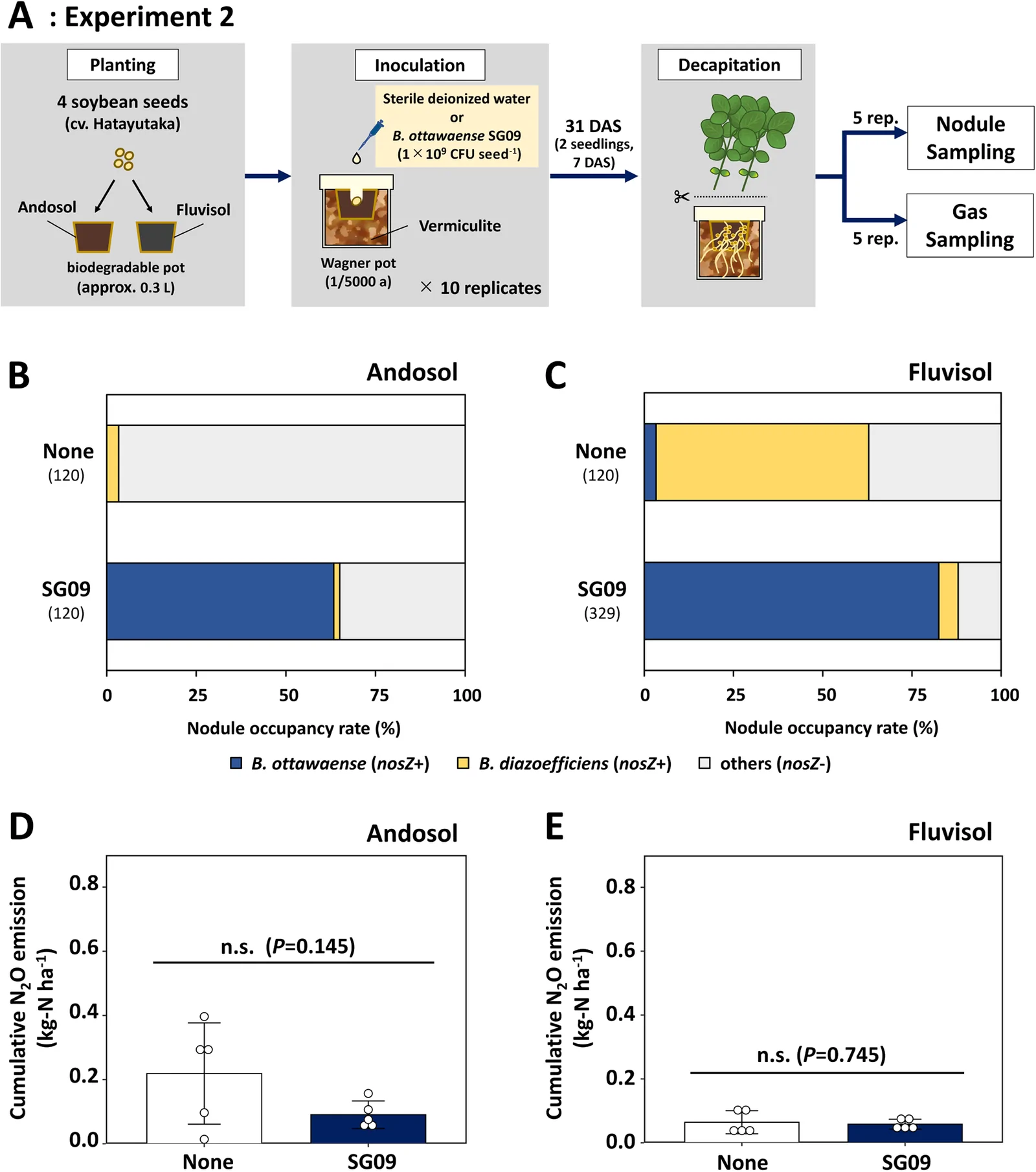
Effects of bradyrhizobial inoculations with two field soils in Experiment 2. (A) Overview of Experiment 2. Treatments are non-inoculation (None) or an inoculation with *Bradyrhizobium ottawaense* SG09 in Andosol or Fluvisol. (B, C) Nodule occupancy rates of *nosZ*+ bradyrhizobia in (B) Andosol and (C) Fluvisol. (*n*=3 replicate pots). The total number of analyzed nodules is shown in parentheses under each treatment label. More detailed nodule occupancy rates are shown in Fig. S3. (D, E) Cumulative N_2_O emissions from (D) Andosol and (E) Fluvisol for 20 days from shoot decapitation. Values are means±SD (*n*=5 replicate pots). ‘n.s.’ means no significant difference (*P*>0.05); the significance of differences was evaluated using the *t*-test.

**Fig. 3.**
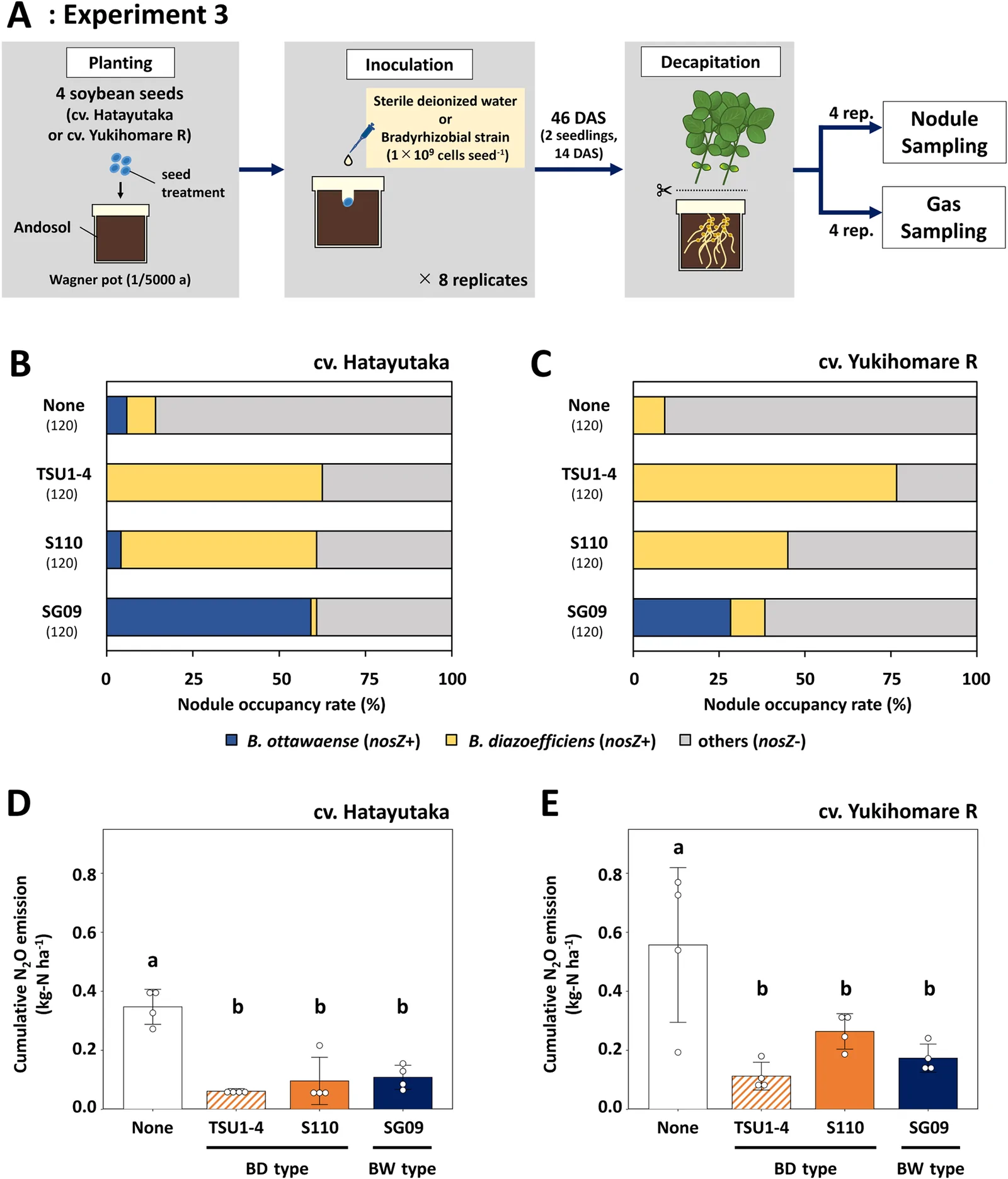
Effects of bradyrhizobial inoculations with two soybean cultivars in Experiment 3. (A) Overview of Experiment 3. Treatments for Experiment 3 were non-inoculation (None), an inoculation with *Bradyrhizobium diazoefficiens* TSU1-4 or the S110 group, or an inoculation with *Bradyrhizobium ottawaense* SG09 for ‘Hatayutaka’ or ‘Yukihomare R’. (B, C) Nodule occupancy rates of *nosZ*+ bradyrhizobia in nodules of (B) ‘Hatayutaka’ and (C) ‘Yukihomare R’. (*n*=4 replicate pots). (D, E) Cumulative N_2_O emissions from Andosol with nodules of (D) ‘Hatayutaka’ and (E) ‘Yukihomare R’ for 45 days after shoot decapitation. Values are means±SD (*n*=4 replicate pots). Different characters indicate significant differences (*P*<0.05); the significance of differences was evaluated using Tukey’s HSD test.

**Table 1.** Bradyrhizobial strains used in this study

Strain name^*1^	Species	Isolation site	Host plant	*nosZ*	Nodulation^*2^	Accession No. (genome)	Reference
TSU1-4 (TS1-4)	*Bradyrhizobium diazoefficiens*	Tsukuba, Ibaraki	Soybean	+	+	—	Shiina *et al.*, 2014; Hara *et al.*, 2024; Toyoda *et al.*, 2024,
SF21	*Bradyrhizobium ottawaense*	Nihonmatsu, Fukushima	Sorghum	+	+	SAMD00634907	Wasai-Hara *et al.*, 2023
SG09	*Bradyrhizobium ottawaense*	Nihonmatsu, Fukushima	Sorghum	+	+	SAMD00179679	Wasai-Hara *et al.*, 2020; 2023
SG10	*Bradyrhizobium ottawaense*	Nihonmatsu, Fukushima	Sorghum	+	+	SAMD00634909	Wasai-Hara *et al.*, 2020; 2023
SG23	*Bradyrhizobium ottawaense*	Nihonmatsu, Fukushima	Sorghum	+	+	SAMD00634900	Wasai-Hara *et al.*, 2020; 2023
SH12	*Bradyrhizobium ottawaense*	Nihonmatsu, Fukushima	Sorghum	+	+	SAMD00634901	Wasai-Hara *et al.*, 2023
FA1	*Bradyrhizobium ottawaense*	Numata, Gunma	Soybean	+	+	—	Itakura *et al.*, 2026
FY1	*Bradyrhizobium ottawaense*	Numata, Gunma	Soybean	+	+	—	Itakura *et al.*, 2026
FY2	*Bradyrhizobium ottawaense*	Numata, Gunma	Soybean	+	+	—	Nishida *et al.*, 2025; Itakura *et al.*, 2026
S110: bacterial mixture of 10 strains of *B. diazoefficiens*							
ZK1	*Bradyrhizobium diazoefficiens*	Kamishihoro, Hokkaido	Soybean	+	+	SAMD01705412	This study
ZK4	*Bradyrhizobium diazoefficiens*	Kamishihoro, Hokkaido	Soybean	+	+	SAMD01705413	This study
ZK7	*Bradyrhizobium diazoefficiens*	Kamishihoro, Hokkaido	Soybean	+	+	SAMD01705414	This study
ZK12	*Bradyrhizobium diazoefficiens*	Kamishihoro, Hokkaido	Soybean	+	+	SAMD01705415	This study
ZK14	*Bradyrhizobium diazoefficiens*	Kamishihoro, Hokkaido	Soybean	+	+	SAMD01705416	This study
ZK17	*Bradyrhizobium diazoefficiens*	Kamishihoro, Hokkaido	Soybean	+	+	SAMD01705417	This study
ZK18	*Bradyrhizobium diazoefficiens*	Kamishihoro, Hokkaido	Soybean	+	+	SAMD01705418	This study
ZK19	*Bradyrhizobium diazoefficiens*	Kamishihoro, Hokkaido	Soybean	+	+	SAMD01705419	This study
ZK23	*Bradyrhizobium diazoefficiens*	Kamishihoro, Hokkaido	Soybean	+	+	SAMD01705420	This study
ZK25	*Bradyrhizobium diazoefficiens*	Kamishihoro, Hokkaido	Soybean	+	+	SAMD01705421	This study

*1 Sequence identities between strains of *Bradyrhizobium diazoefficiens* and *Bradyrhizobium ottawaense* were similar, according to a phylogenetic anal­ysis based on 16S–23S rRNA intergenic transcribed spacer (ITS) region sequences (Fig. S1). *2 Nodulation ability was evaluated using an inoculation test with soybean.

**Table 2.** Nodule occupancy rate by *nosZ*-possessing bradyrhizobia and cumulative N_2_O emissions in each experiment.

Experiment No.	Treatment			Soil type	Soybean cultivar	Nodule occupancy by *nosZ* PCR (%)^*1^				Cumulative N_2_O emission (g-N ha^–1^)^*1^		N_2_O reduction rate (%)^*2^
	Inoculated strain	*nosZ* type of bradyrhizobia				BD type (%)		BW type (%)				
Experiment 1	None (control)	—	—	Andosol	Hatayutaka	1.6±1.9	b	4.0±6.1	b	90.0±51.0	a	—
	TSU1-4	*nosZ+*	BD-type	Andosol	Hatayutaka	92.8±4.9	a	0.0±0.0	b	16.9±7.8	b	81.3
	SF21	*nosZ+*	BW-type	Andosol	Hatayutaka	0.0±0.0	b	82.3±12.5	a	14.1±15.6	b	84.3
	SG09	*nosZ+*	BW-type	Andosol	Hatayutaka	0.8±1.6	b	80.6±8.7	a	18.3±12.2	b	79.7
	SG10	*nosZ+*	BW-type	Andosol	Hatayutaka	0.0±0.0	b	81.5±8.5	a	16.7±8.9	b	81.5
	SG23	*nosZ+*	BW-type	Andosol	Hatayutaka	0.0±0.0	b	90.0±9.5	a	10.9±2.2	b	87.9
	SH12	*nosZ+*	BW-type	Andosol	Hatayutaka	0.0±0.0	b	69.4±12.5	a	25.6±13.7	b	71.5
	FA1	*nosZ+*	BW-type	Andosol	Hatayutaka	0.8±1.6	b	70.2±14.7	a	18.4±13.1	b	79.6
	FY1	*nosZ+*	BW-type	Andosol	Hatayutaka	0.0±0.0	b	80.6±13.7	a	20.1±17.3	b	77.6
	FY2	*nosZ+*	BW-type	Andosol	Hatayutaka	1.6±1.9	b	66.1±17.6	a	21.8±13.0	b	75.8
Experiment 2^#^	None (control)	—	—	Andosol	Hatayutaka	3.3±3.8	n.s.	0.0±0.0	b	218.9±157.8	a	—
	SG09	*nosZ+*	BW-type	Andosol	Hatayutaka	1.7±1.4	n.s.	63.3±11.8	a	90.6±42.9	a	58.6
	None (control)	—	—	Fluvisol	Hatayutaka	59.2±3.8	a	3.3±1.4	b	64.3±36.1	a	—
	SG09	*nosZ+*	BW-type	Fluvisol	Hatayutaka	5.1±3.7	b	83.0±6.3	a	58.3±15.5	a	9.4
Experiment 3	None (control)	—	—	Andosol	Hatayutaka	8.3±8.8	c	5.8±5.7	b	347.4±59.3	a	—
	TSU1-4	*nosZ+*	BD-type	Andosol	Hatayutaka	62.5±19.9	a	0.0±0.0	b	61.3±8.7	b	82.4
	S110	*nosZ+*	BD-type	Andosol	Hatayutaka	56.7±7.2	b	4.2±1.7	b	96.2±80.2	b	72.3
	SG09	*nosZ+*	BW-type	Andosol	Hatayutaka	1.7±3.4	c	59.2±14.2	a	108.6±40.9	b	68.7
	None (control)	—	—	Andosol	Yukihomare R	9.2±6.9	c	0.0±0.0	b	557.0±262.3	a	—
	TSU1-4	*nosZ+*	BD-type	Andosol	Yukihomare R	76.7±20.2	a	0.0±0.0	b	112.5±47.0	b	79.8
	S110	*nosZ+*	BD-type	Andosol	Yukihomare R	45.0±12.9	b	0.0±0.0	b	264.0±60.1	b	52.6
	SG09	*nosZ+*	BW-type	Andosol	Yukihomare R	10.0±9.8	c	28.3±25.7	a	173.4±47.7	b	68.9

*1 Values are means±SD (nodule occupancy rate: *n*=4 or 3^#^, cumulative N_2_O emission: *n*=4 or 5^#^). Nodule occupancy rates by *Bradyrhizobium diazoefficiens* (BD type) or *Bradyrhizobium ottawaense* (BW type) and cumulative N_2_O emissions were statistically evaluated using Tukey’s HSD test (Experiments 1 and 3) or the *t*-test (Experiment 2). Different letters indicate significant differences (*P*<0.05). *2 N_2_O reduction rate (%)=(1–(total cumulative N_2_O emissions in the inoculated treatment)/(total cumulative N_2_O emissions in the non-inoculated treatment))×100 Total cumulative N_2_O emissions represent the sum across all replicates in each treatment.
