# Supplementary material for: Inoculation with Bradyrhizobium ottawaense Effectively Reduces N2O Emissions from Degraded Soybean Nodules in Andosols Dominated by nosZ-lacking Bradyrhizobia
Source: Microbes Environ. 2026 Sep 10;41(3):ME26022. doi: 10.1264/jsme2.ME26022 (PMC13659342; doi:10.1264/jsme2.ME26022)
Supplement: Supplementary file 1 [file 41_26022_s1.pdf]

Supplementary Materials

Inoculation with *Bradyrhizobium ottawaense* Effectively Reduces N_2O Emissions from Degraded Soybean Nodules in Andosols Dominated by *nosZ*-lacking bradyrhizobia

Misa Tsubokura[1], Shintaro Hara[1†], Tsubasa Ohbayashi[1], Masanori Takeda[1], Yuma Sasaki[1], Haruka Ukita[1§], Miho Fujimori[1§], Kaori Kakizaki[2], Manabu Itakura[2], Kiwamu Minamisawa[2], Hiroko Akiyama[1,*]

1 Institute for Agro-Environmental Sciences, National Agriculture and Food Research Organization (NARO), Tsukuba, Ibaraki, 305-8604, Japan

2 Graduate School of Life Sciences, Tohoku University, Sendai, Miyagi, 980–8577, Japan

† Present address: Hokkaido Agricultural Research Center, National Agriculture and Food Research Organization (NARO), Sapporo, Hokkaido 062-8555, Japan

§ Present address: Institute of Livestock and Grassland Science, National Agriculture and Food Research Organization (NARO), Tsukuba, Ibaraki, 305-0901, Japan

* Corresponding author:

Hiroko Akiyama: E-mail: akiyama.hiroko593@naro.go.jp; Tel: +81- 29-838-8231

Materials and Methods

Detailed pot experimental designs

Experiment 4 (Supplementary experiment)

Experiment 4 was conducted to evaluate the inoculation effect of *B. ottawaense* using 'Hatayutaka' and 'Satonohohoemi' as in Experiment 3 (**Fig. S7**). 'Satonohohoemi' is a late-maturing commercial cultivar developed for northeastern (Tohoku) regions of Japan. The pot experimental procedure was basically performed as described in Experiment 3. The inoculation effect of *B. ottawaense* SG09 was compared with that of the *B. diazoefficiens* S110 group. Each treatment was performed in eight replicates. Thinning and shoot decapitation were done at 14 DAS and 34 DAS, respectively. Gas sampling was conducted for 30 days after decapitation in the greenhouses.

Plant growth conditions

All experiments were conducted in the greenhouses at NIAES. Experiments 1-1, 1-2, 2, 3, and 4 were conducted during July 12–August 18, 2022, April 12–May 23, 2022, April 27–May 28, 2021, March 23–May 8, 2023, and December 7, 2023–January 10, 2024, respectively. The temperature in the greenhouses was set at 25°C/20°C (16 h-day/8 h-night) in Experiments 1, 3 and 4, or at 28°C/26°C (12 h-day/12 h-night) in Experiment 2. In Experiments 3 and 4, supplemental LED lighting was used to prevent spindly shoot growth. Soybean plants were cultivated without fertilizer application. Watering was performed every day using a wick in Experiments 1 and 2 or an automatic watering device with a timer in Experiments 3 and 4.

Measurement of plant growth parameters

After shoot decapitation, soybean shoot parts were dried at 80ºC for over 72 h in Experiments 1-2, 3, and 4, or at 70ºC for over 96 h in Experiment 2, and then the dry weight was measured. Subsequently, in Experiment 2, the nitrogen contents of the dried soybean plants were analyzed using a CN analyzer (NC-22F, SCAS, Japan).

Monitoring of soil water content in Experiments 3 and 4

The soil volumetric water contents were monitored in five pots using a soil moisture sensor (EC-5, METER Group, Inc., Pullman, WA, USA) from decapitation to the final date of gas sampling. The soil volumetric water content values were converted to the water-filled pore space (WFPS) using a soil porosity value (0.76) and an empirical calibration curve for Andosol ($y = 0.95x+0.086$, $R^2 = 0.99$) (**Akiyama *et al*., 2014**). The porosity was calculated using the measured bulk density ($0.65\ g\ cm^{-3}$) and particle density ($2.65\ g\ cm^{-3}$).

Statistical analysis

Statistical analyses of nodule occupancy rate, cumulative N_2O emission, nodule number, nodule fresh weight, shoot dry weight, and shoot nitrogen content were performed using R software (version 4.3.1) (**R Core Team, 2023**). A two-way analysis of variance (ANOVA) was conducted using R software. In Experiment 2, cumulative N_2O emissions were analyzed using soil type (Andosol or Fluvisol) and bradyrhizobial inoculation (None or SG09) as factors. In Experiment 3, nodule number was analyzed using soybean cultivar ('Hatayutaka' or 'Yukihomare R') and bradyrhizobial inoculation (None, TSU1-4, S110 group or SG09) as factors. Before conducting the two-way ANOVA, the residual normality and homogeneity of variance were evaluated using the Shapiro-Wilk test and the Brown–Forsythe test or the Bartlett test in R software, respectively.

Supplementary Figures

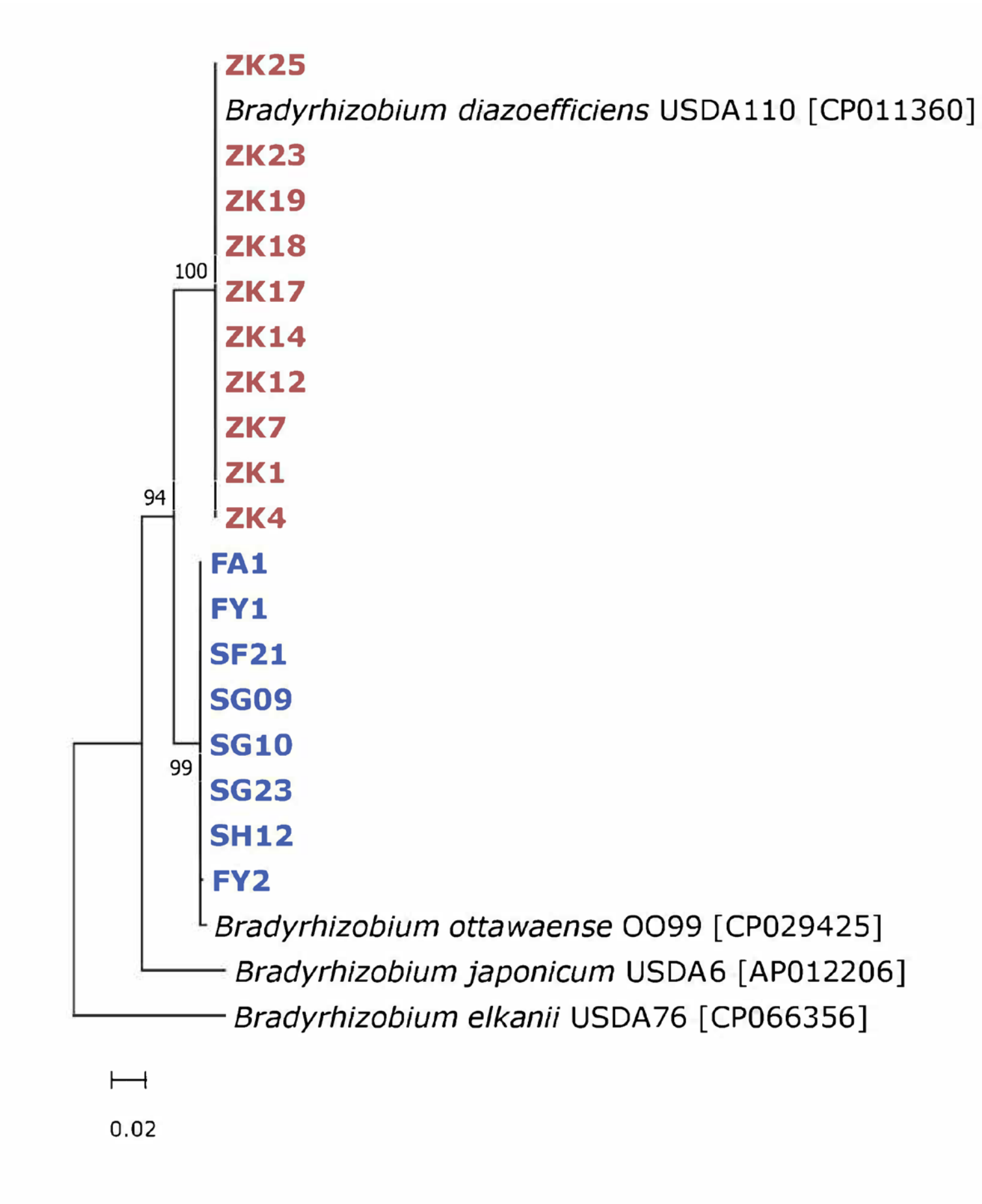

Fig. S1. Phylogenetic tree of bradyrhizobial strains based on the internal transcribed spacer (ITS) region between the 16S and 23S rRNA genes.

This phylogenetic tree shows that eight strains of *B. ottawaense* and ten strains belonging to the S110 group of *B. diazoefficiens* formed a monophyletic group, respectively.

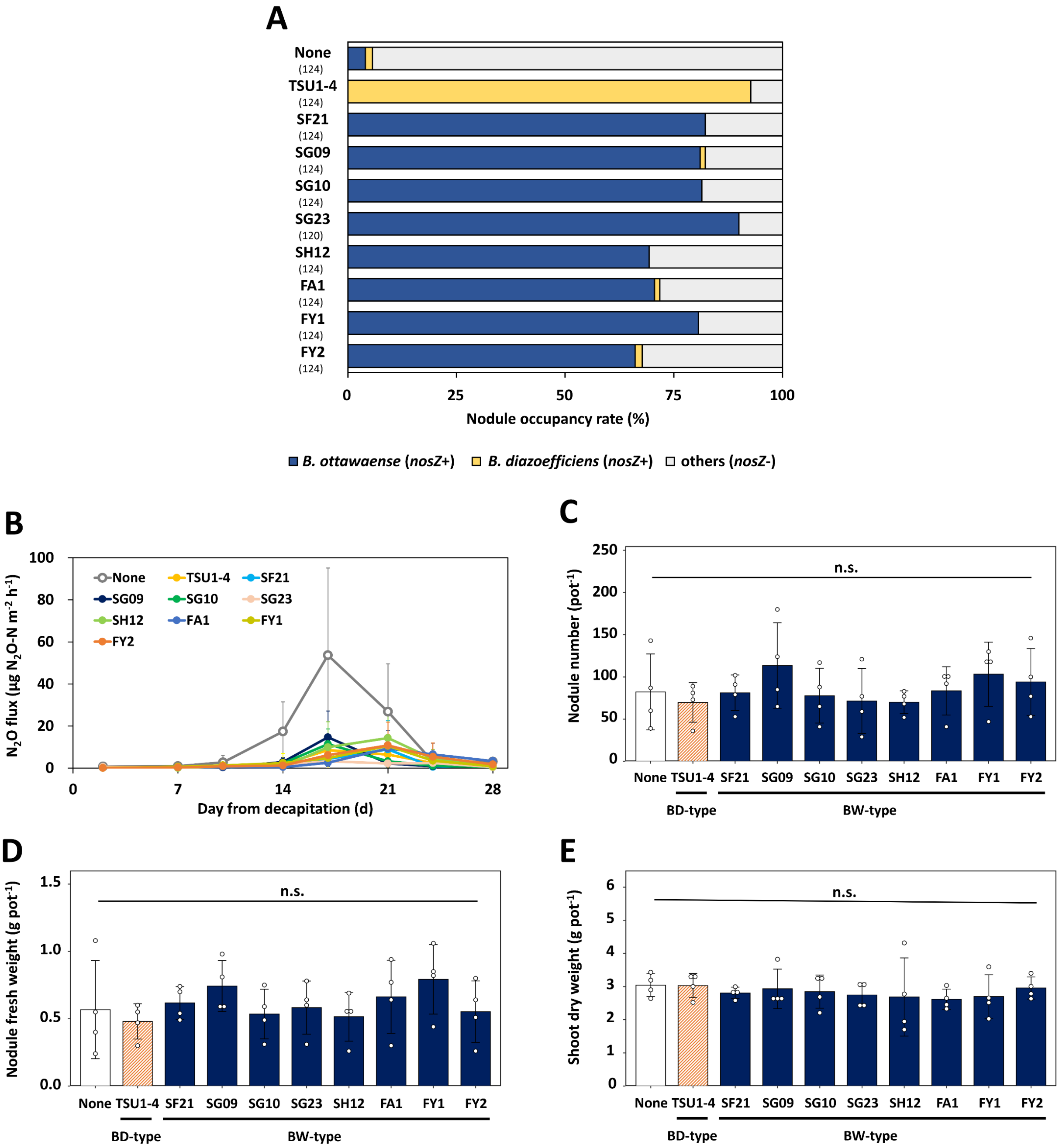

Fig. S2. Effects of bradyrhizobial inoculation on N_2O emission and plant growth in Experiment 1.

(A) Nodule occupancy rates by *nosZ*+ bradyrhizobia in Experiment 1-1. Nodule occupancy rates of *B. ottawaense* and *B. diazoefficiens* were analyzed by *nosZ* PCR (n = 4 replicate pots). Total number of analyzed nodules is shown in parentheses under each treatment label. (B) Temporal changes in N_2O fluxes from Andosol after shoot decapitation in Experiment 1-2. Values are means ± SD (n = 4 replicate pots). (C, D) (C) Nodule number and (D) nodule fresh weight at 37 days after sowing for nodule occupancy analysis in Experiment 1-1. (E) Shoot dry weight of at 41 days after sowing for N_2O emission analysis in Experiment 1-2. "BD-type" and "BW-type" indicate "*Bradyrhizobium diazoefficiens*" and "*Bradyrhizobium ottawaense*", respectively. Values are means ± SD (n = 4 replicate pots). 'n.s.' means no significant difference using Tukey's HSD test (p > 0.05).

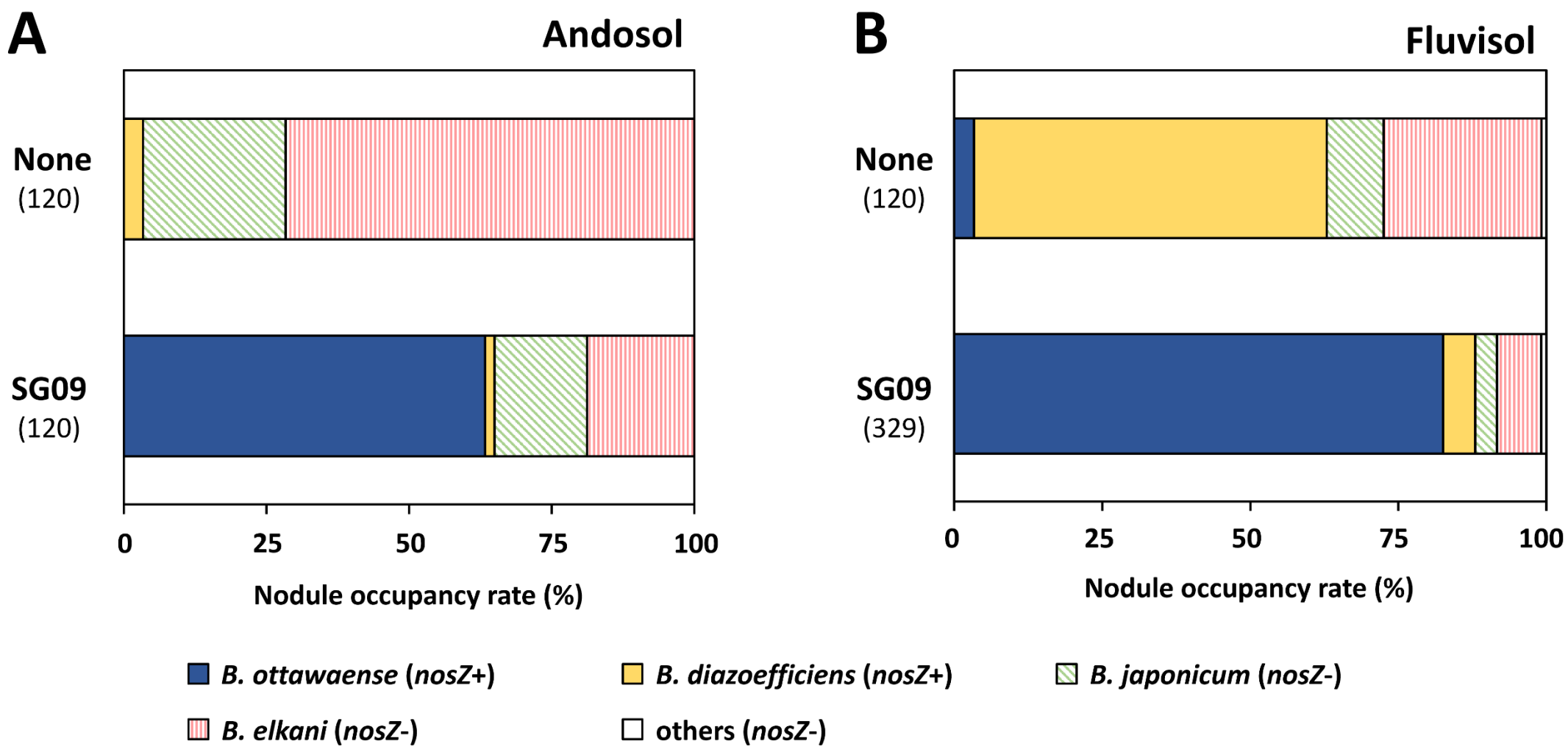

Fig. S3. Bradyrhizobial diversity in nodules of soybean cultivated in (A) Andosol and (B) Fluvisol in Experiment 2.

Nodule occupancy rates of the four main bradyrhizobia species were analyzed using ITS-RFLP (n = 3 replicate pots). Total number of analyzed nodules is shown in parentheses under each treatment label.

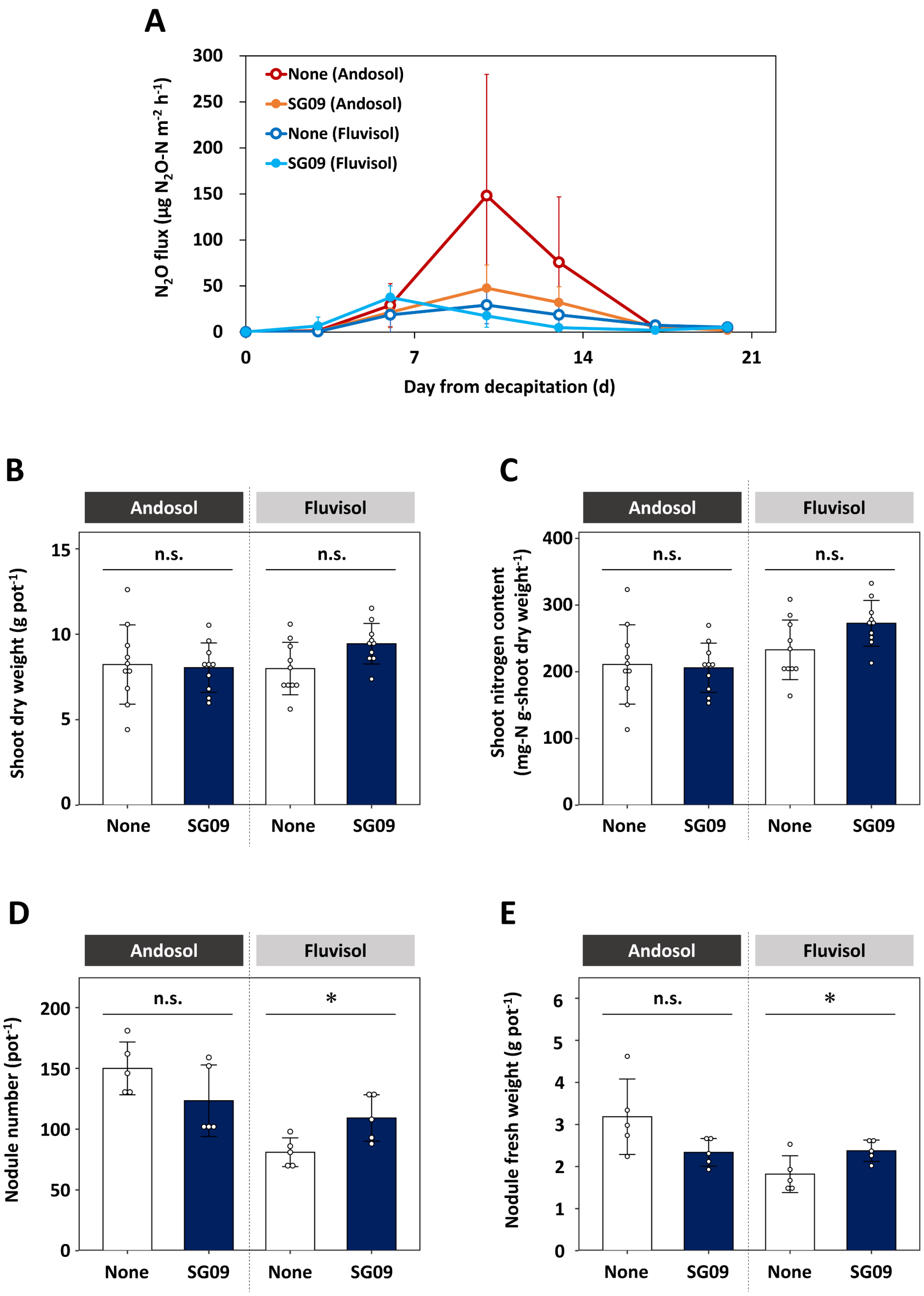

Fig. S4. Effects of inoculation with *Bradyrhizobium ottawaense* SG09 on N_2O emissions and plant growth in Experiment 2.

(A) Temporal changes in N_2O fluxes from Andosol and Fluvisol after shoot decapitation. Values are means ± SD (n = 5 replicate pots). (B–E) (B) Shoot dry weight, (C) shoot nitrogen content, (D) nodule number, and (E) nodule fresh weight at the date of shoot decapitation. Values are means ± SD (shoot dry weight and nitrogen content: n = 10 replicate pots, nodule number and nodule fresh weight: n = 5 replicate pots). 'n.s.' means no significant difference ($p > 0.05$) and asterisk indicates significant differences using t-test ($p < 0.05$).

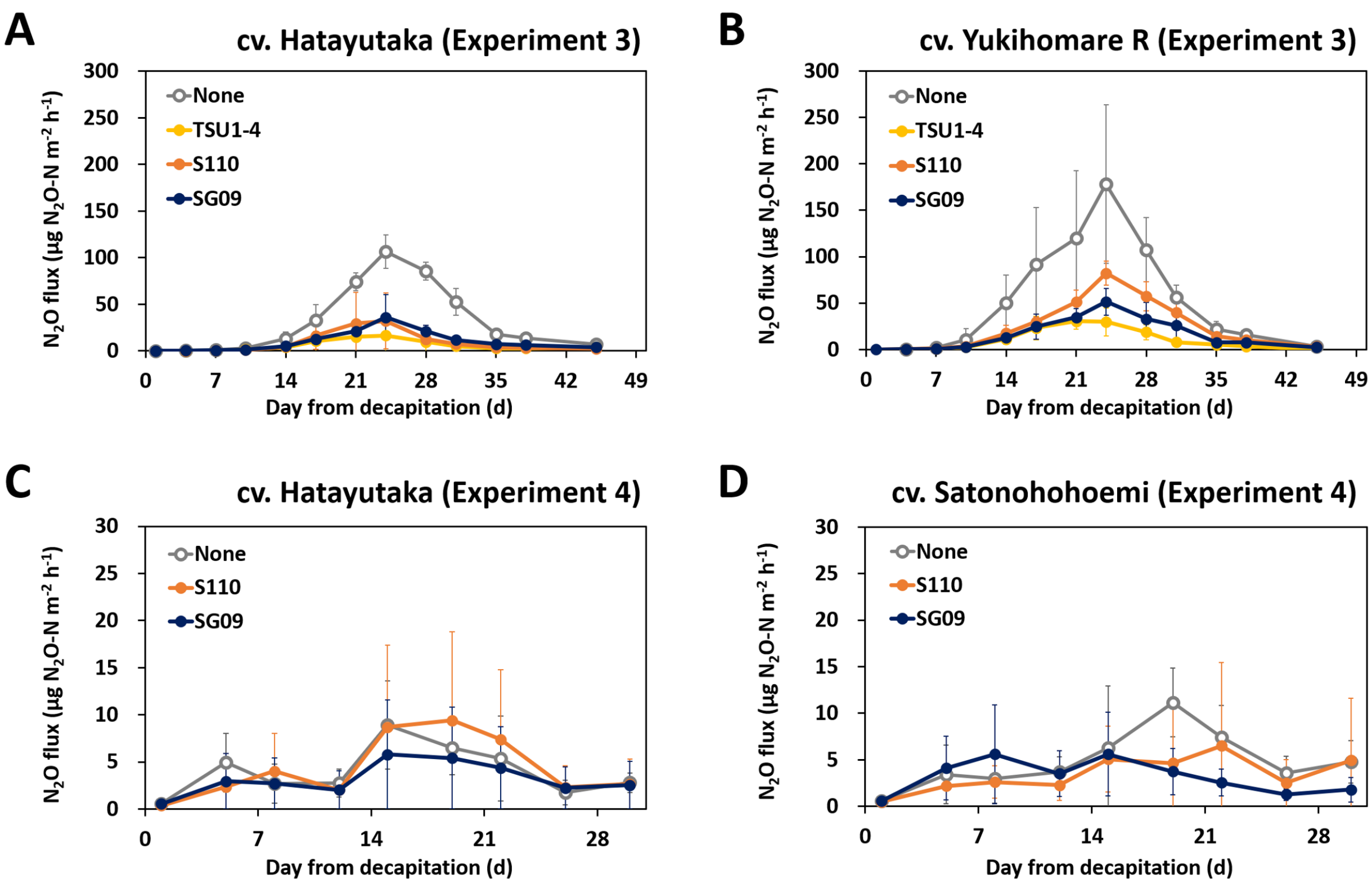

Fig. S5. Temporal changes in N_2O fluxes from Andosol with nodules of (A) 'Hatayutaka' and (B) 'Yukihomare R' in Experiment 3, and (C) 'Hatayutaka' and (D) 'Satonohohoemi' in Experiment 4.

Values are means ± SD (n = 4 replicate pots).

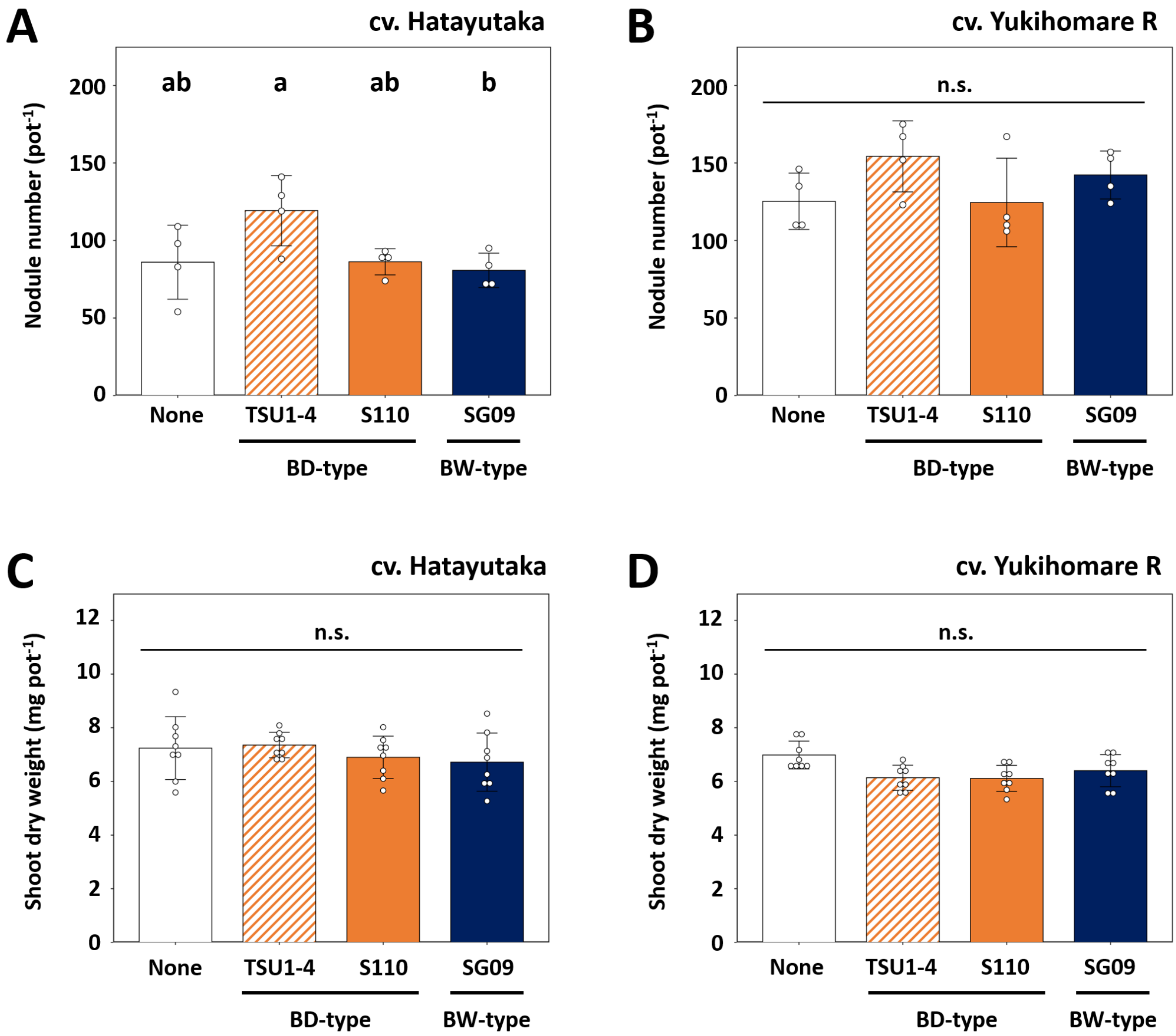

Fig. S6. Effects of the bradyrhizobial inoculation on plant growth in Experiment 3.

(A, B) Nodule number of (A) 'Hatayutaka' and (B) 'Yukihomare R' at the date of decapitation. (C, D) Shoot dry weight of (C) 'Hatayutaka' and (D) 'Yukihomare R' at the date of decapitation. "BD-type" and "BW-type" indicated "*Bradyrhizobium diazoefficiens*" and "*Bradyrhizobium ottawaense*", respectively. Values are means ± SD (nodule number: $n = 4$ replicate pots, shoot dry weight: $n = 8$ replicate pots). Different characters indicate significant differences using Tukey's HSD test ($p < 0.05$).

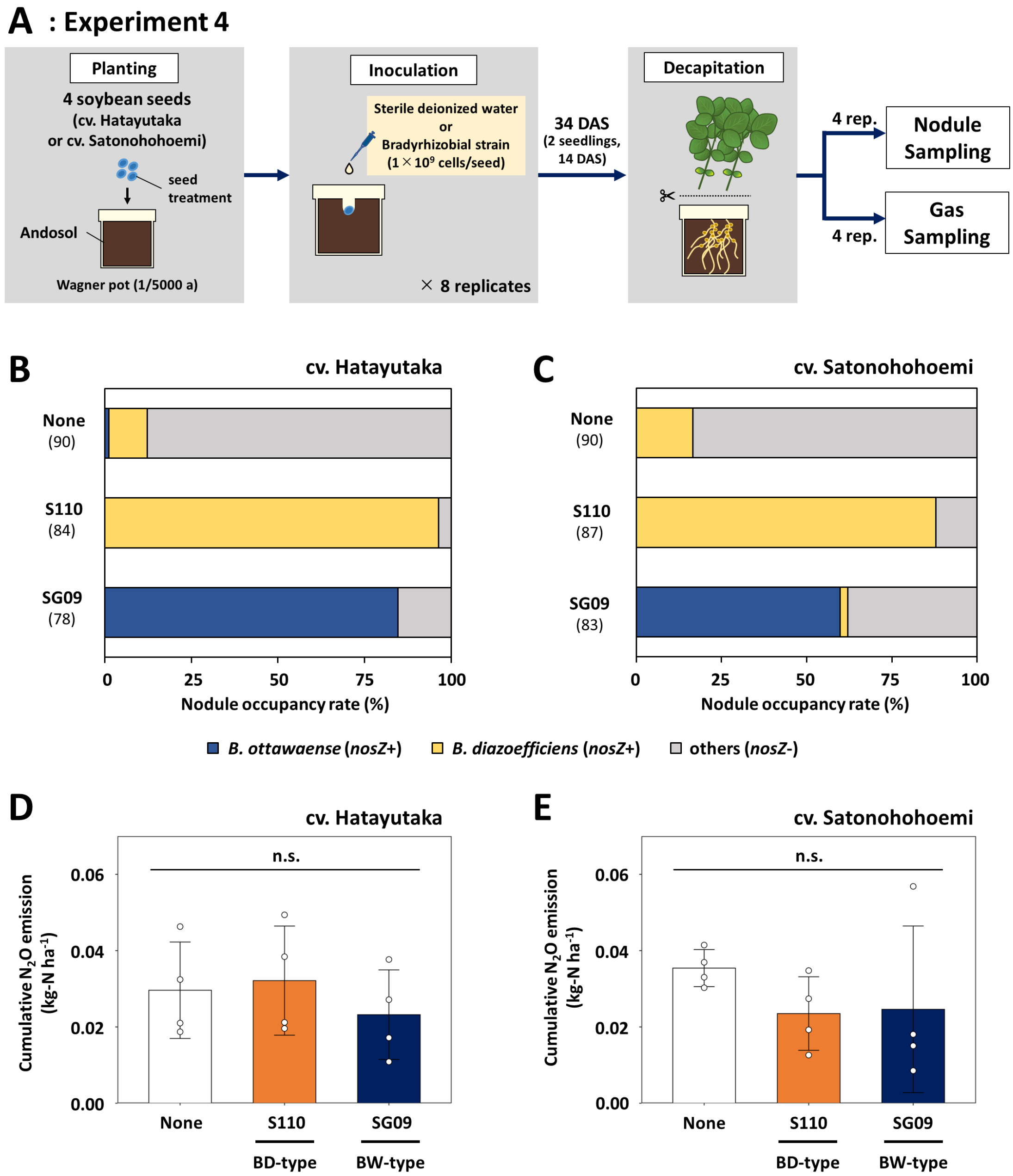

Fig. S7. Effects of bradyrhizobial inoculation with two soybean cultivars in Experiment 4.

(A) Overview of Experiment 4. Treatments for Experiment 4 were non-inoculation (None), inoculation with *B. diazoefficiens* S110 group or *B. ottawaense* SG09 for 'Hatayutaka' or 'Satonohohoemi'. DAS: days after seedling. (B, C) Nodule occupancy rates of *nosZ*+ bradyrhizobia in nodules of (B) 'Hatayutaka' and (C) 'Satonohohoemi'. Nodule occupancy rates of *B. ottawaense* and *B. diazoefficiens* were analyzed by *nosZ* PCR ($n = 4$ replicate pots). Total number of analyzed nodules is shown in parentheses under each treatment label. (D, E) Cumulative N_2O emissions from Andosol with nodules of (D) 'Hatayutaka' and (E) 'Satonohohoemi' for 30 days after shoot decapitation. "BD-type" and "BW-type" indicate "*Bradyrhizobium diazoefficiens*" and "*Bradyrhizobium ottawaense*", respectively. Values are means ± SD ($n = 4$ replicate pots). Different characters indicate significant differences ($p < 0.05$); statistical significance was evaluated using Tukey's HSD test.

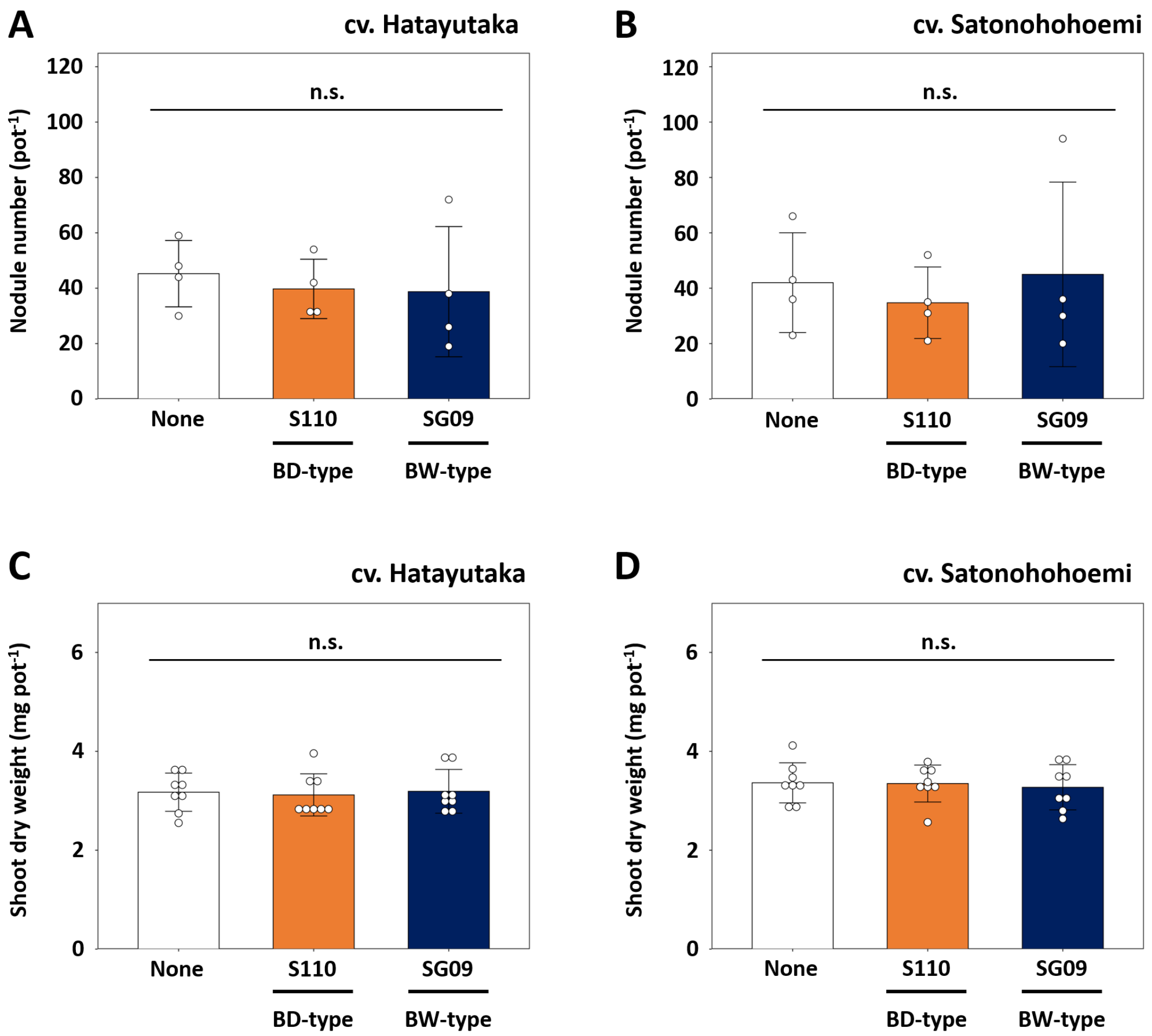

Fig. S8. Effects of bradyrhizobial inoculation on plant growth in Experiment 4.

(A, B) Nodule number of (A) 'Hatayutaka' and (B) 'Satonohohoemi'. (C, D) Shoot dry weight of (C) 'Hatayutaka' and (D) 'Satonohohoemi' at the date of decapitation. "BD-type" and "BW-type" indicated "*Bradyrhizobium diazoefficiens*" and "*Bradyrhizobium ottawaense*", respectively. Values are means ± SD (nodule number: n = 4 replicate pots, shoot dry weight: n = 8 replicate pots). Different characters indicate significant differences using Tukey's HSD test ($p < 0.05$).

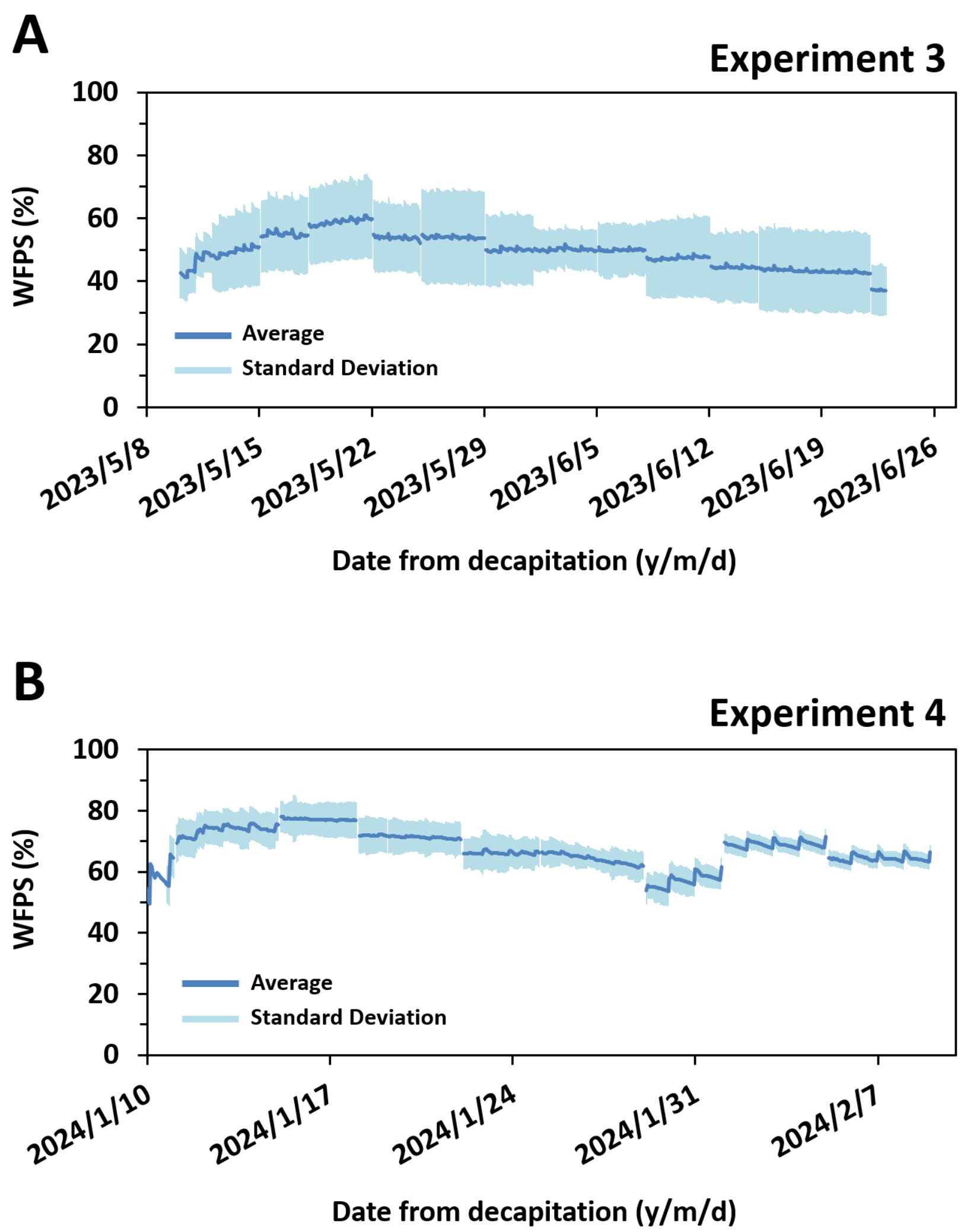

Fig. S9. Temporal changes in water-filled pore space (WFPS) at a depth of 5 cm after decapitation of soybean plant in (A) Experiment 3 and (B) Experiment 4.

Values are means ± SD (Experiment 3: n = 5 replicate pots, Experiment 4: n = 6 replicate pots).

Table S1. Primer sets for nodule analysis

Target gene / region	Primer name	sequence (5' to 3')	References
nosZ (*Bradyrhizobium diazoefficiens*)	nosZ_BD-F	GACGGCGATACCATGAAAGT	Itakura *et al*., 2013
	nosZ_BD-R	TCGGGTTGATCTTGGAACGG	Hara *et al*., 2024
nosZ (*Bradyrhizobium ottawaense*)	nosZ_BW-F	CAAGCCCGAGAATGATCAGC	Hara *et al*., 2024
	nosZ_BW-R	CCGGCCCTTCATCTCCATAT	Hara *et al*., 2024
16S–23S rDNA ITS region (*Bradyrhizobium* spp.)	BraITS-F	GACTGGGGTGAAGTCGTAAC	Saeki *et al*., 2006
	BraITS-R	ACGTCCTTCATCGCCTC	Saeki *et al*., 2006

Table S2. Nodule occupancy rate by *nosZ*-possessing bradyrhizobia and cumulative N_2O emissions in Experiment 4.

Treatment			Soil type	Soybean cultivar	Nodule occupancy by *nosZ* PCR (%)[*1]				Cumulative N_2O emission (mg-N ha^{-1})[*1]		N_2O reduction rate (%)[*2]
Inoculated strain	*nosZ* type of bradyrhizobia				BD-type (%)		BW-type (%)				
None (control)	-	-	Andosol	Hatayutaka	11.1 ± 11.7	b	1.1 ± 1.9	b	29.6 ± 12.6	a	-
S110	*nosZ*+	BD-type	Andosol	Hatayutaka	96.5 ± 3.3	a	0.0 ± 0.0	b	32.1 ± 14.3	a	−8.5
SG09	*nosZ*+	BW-type	Andosol	Hatayutaka	0.0 ± 0.0	b	84.5 ± 10.2	a	23.2 ± 11.7	a	21.6
None (control)	-	-	Andosol	Satonohohoemi	0.0 ± 0.0	b	16.7 ± 12.0	b	35.4 ± 4.9	a	-
S110	*nosZ*+	BD-type	Andosol	Satonohohoemi	88.6 ± 13.2	a	0.0 ± 0.0	b	23.5 ± 9.6	a	33.7
SG09	*nosZ*+	BW-type	Andosol	Satonohohoemi	2.2 ± 3.9	b	60.2 ± 13.7	a	24.6 ± 21.8	a	30.5

*1 Values are means ± SD ($n = 4$). Nodule occupancy rates by *Bradyrhizobium diazoefficiens* (BD-type) or *Bradyrhizobium ottawaense* (BW-type) and cumulative N_2O emissions were statistically evaluated using Tukey's HSD test. Different letters indicate significant differences ($p < 0.05$).

*2 N_2O reduction rate (%) = (1−(total cumulative N_2O emissions in the inoculated treatment) / (total cumulative N_2O emissions in the non-inoculated treatment)) × 100

Noted that total cumulative N_2O emissions represent the sum across all replicates.

Table S3. Cumulative sunshine duration and solar radiation in each experimental period.

Experiment No.	Cumulative sunshine duration (h)	Cumulative solar radiation (MJ/m^2)	Cultivation period（day)
Experiment 1-1<br>(for analyzing nodule occupancy)	222.2	672.4	37 [July 12th–August 18th in 2022]
Experiment 1-2<br>(for analyzing N_2O emission)	206.0	648.3	41 [April 12th–May 23rd in 2022]
Experiment 2	152.3	492.2	31 [April 27th–May 28th in 2021]
Experiment 3	303.2	797.0	46 [March 23th–May 8th in 2023]
Experiment 4	158.9	306.0	34 [December 7th in 2023–January 10th in 2024]

Data were obtained from Weather Data Acquisition System of NARO (Station name: Kannondai,Tsukuba).

References

Akiyama, H., Morimoto, S., Tago, K., Hoshino, Y.T., Nagaoka, K., Yamasaki, M., *et al*. (2014) Relationships between ammonia oxidizers and N_2O and CH_4 fluxes in agricultural fields with different soil types. *Soil Sci Plant Nutr* **60**: 520–529.

Hara, S., Kakizaki, K., Bamba, M., Itakura, M., Sugawara, M., Suzuki, A., et al. (2024) Does rhizobial inoculation change the microbial community in field soils? A comparison with agricultural land-use changes. *Microbes Environ* **39**: ME24006.

Itakura, M., Uchida, Y., Akiyama, H., Hoshino, Y.T., Shimomura, Y., Morimoto, S., et al. (2013) Mitigation of nitrous oxide emissions from soils by *Bradyrhizobium japonicum* inoculation. *Nat Clim Chang* **3**: 208–212.

R Core Team (2023) R: A language and environment for statistical computing. Vienna, Austria: R Foundation for Statistical Computing. URL https://www.R-project.org/

Saeki, Y., Aimi, N., Tsukamoto, S., Yamakawa, T., Nagatomo, Y., and Akao, S. (2006) Diversity and geographical distribution of indigenous soybean-nodulating bradyrhizobia in Japan. *Soil Sci Plant Nutr* **52**: 418–426.
